# Disaster mitigation based on Minangkabau indigenous knowledge in social studies learning

**DOI:** 10.4102/jamba.v18i1.2047

**Published:** 2026-06-09

**Authors:** Sri Rahayu, Faishal Yasin, Ade Pratama, Rinel Fitlayeni, Annissa Amini, Maulana A. Pratama

**Affiliations:** 1 Department of Social Studies Education, Faculty of Social Science and Humanities, Universitas PGRI Sumatera Barat, Padang, Indonesia; 2 Department of Sociology Education, Faculty of Social Science and Humanities, Universitas PGRI Sumatera Barat, Padang, Indonesia; 3 Department of Information Technology, Faculty of Science and Technology, Universitas PGRI Sumatera Barat, Padang, Indonesia

**Keywords:** disaster mitigation, indigenous knowledge, Minangkabau, social studies learning, culture-based education

## Abstract

Indonesia ranks among the world’s most disaster-prone countries due to its location within the Pacific Ring of Fire. West Sumatra, in particular, frequently experiences earthquakes, tsunamis, volcanic eruptions, flash floods, and hydrometeorological hazards. These recurring disasters highlight the urgent need for mitigation strategies that integrate scientific approaches with traditional indigenous knowledge. This study explores the potential of Minangkabau indigenous knowledge as a source of values, knowledge, and socio-cultural practices that can be incorporated into social studies (IPS) learning to enhance students’ disaster literacy. Using a qualitative descriptive-analytical design, data were collected through a literature review, classroom observations, in-depth interviews with teachers, students, traditional leaders, and disaster management officials, and documentation of curricula, lesson plans, and cultural practices. The data were analysed using Miles and Huberman’s interactive model through reduction, display, and verification. Findings reveal that Minangkabau indigenous knowledge encompasses technical, social, spiritual, and ecological dimensions. Technically, *Rumah Gadang* architecture embodies earthquake-resistant principles through its flexible wooden-peg construction. Socially, oral traditions transmit collective memories of past disasters to strengthen community preparedness. Spiritually, proverbs such as *alam takambang jadi guru* [nature as the ultimate teacher] nurture ecological awareness and moral reflection. Ecologically, the *rimbo larangan* [sacred forest] system supports environmental sustainability and flood prevention. Integrating these dimensions into social studies through project-based learning, folklore documentation, and culture-based disaster simulations enhances students’ disaster literacy while preserving cultural identity.

**Contribution:**

This study contributes to disaster education by linking traditional ecological knowledge with modern mitigation science and proposing a culture-based learning model that promotes resilience, environmental empathy, and sustainability aligned with the Sendai Framework and UNESCO’s Education for Sustainable Development goals.

## Introduction

Natural hazards occur when the balance among natural components is disrupted, leading to environmental instability and large-scale destruction. Indonesia, situated at the convergence of three major tectonic plates, the Indo-Australian Plate moving northward, the Eurasian Plate moving southward, and the Pacific Plate moving westward, is among the most disaster-prone countries in the world. The collision and subduction of these plates exert immense pressure on the Earth’s crust, creating mountainous morphology and uneven topography across the Indonesian archipelago (Rakuasa & Pakniany 2024). Furthermore, Indonesia’s position within the Pacific Ring of Fire, encircled by a chain of active volcanoes, results in frequent occurrences of earthquakes, volcanic eruptions, tsunamis, and other geohazards (Hutchings & Mooney 2021; Uekusa 2017). Among the nation’s provinces, West Sumatra is one of the most vulnerable regions to natural hazard. Data from the National Disaster Management Agency (BNPB) indicate that over the past two decades, the province has faced several major catastrophes. The first was the Padang earthquake on 30 September 2009 (magnitude 7.6), which claimed over 1100 lives, injured thousands, and destroyed tens of thousands of houses and public facilities. The second was the Mentawai tsunami on 25 October 2010, triggered by a 7.2-magnitude earthquake, which devastated coastal areas of the North and South Pagai Islands, South Sipora, and Sikakap, resulting in 509 deaths and hundreds of injuries. More recently, the eruption of Mount Marapi on 17 December 2023, caused 23 fatalities, followed by a cold lava flood (*galodo*) on 11 May 2024, which killed 61 people across Tanah Datar, Agam, Padang Panjang, Padang, and Padang Pariaman Regencies, with several still missing. Earlier that year, flash floods struck Pesisir Selatan (13 March 2024) and spread across 12 districts, displacing thousands (Ayuningtyas et al. 2021). These recurring disasters clearly indicate that the people of West Sumatra live under a persistent threat, necessitating a community culture of vigilance and preparedness. In this context, the Sendai Framework for Disaster Risk Reduction (2015–2030) underscores the importance of leveraging traditional and indigenous knowledge, as well as education as integral components of disaster mitigation (United Nations Office for Disaster Risk Reduction 2015). Similarly, the United Nations Educational, Scientific, and Cultural Organization (UNESCO 2021) advocates for the incorporation of indigenous knowledge into formal education as a means of strengthening community resilience while safeguarding cultural identity (UNESCO 2021). Case studies across Indonesia, such as the among practice in Simeulue, which saved thousands during the 2004 tsunami (Fakhruddin & Elmada 2022), and the Tsunami-Ready model in Bali, which integrates traditional leadership with school participation (Sakya et al. 2023) illustrate the power of local knowledge in protecting communities. The Merdeka Curriculum represents a national curriculum reform policy designed to address the challenges of 21st-century education, including the diversity of social and cultural contexts and the varied learning needs of Indonesian students. This curriculum emphasises the principles of flexibility, differentiated instruction, and character development by granting greater autonomy to schools and educators in designing learning processes that are responsive to students’ characteristics and local environmental contexts (The Ministry of Education, Culture, Research, and Technology of the Republic of Indonesia 2024). One of the defining features of the Merdeka Curriculum is the shift from a content-oriented instructional approach toward a learning paradigm focused on essential competencies and character formation. The curriculum structure is streamlined to provide teachers with broader opportunities for in-depth exploration of subject matter, meaningful learning experiences, and adaptive instructional strategies tailored to students’ learning needs. Within this framework, assessment is no longer positioned solely as a tool for measuring learning outcomes but also functions as a diagnostic instrument to support student-centred learning processes (The Ministry of Education, Culture, Research, and Technology of the Republic of Indonesia 2024). The integration of indigenous knowledge is a strategic component of the Merdeka Curriculum, as it is regarded as a contextual learning resource that bridges academic knowledge and students’ socio-cultural realities. Under *Regulation No. 56 of 2022* of the Ministry of Education, Culture, Research, and Technology, schools are authorised to contextualise the curriculum by incorporating local values, practices, and knowledge into instructional activities. This approach not only enhances the relevance of learning but also contributes to the internalisation of cultural values, the preservation of local identity, and the strengthening of national character. This policy allows schools to include local content reflecting regional characteristics through three mechanisms: (1) integrating indigenous knowledge into existing subjects, (2) embedding it into project-based learning to strengthen the Pancasila Student Profile, and (3) developing indigenous knowledge as an independent subject. The regulation encourages schools to use this flexibility to internalise moral and character values rooted in local culture, thereby enhancing students’ environmental and social awareness (Siska, Sapriya & Febriani 2021). This approach is particularly relevant to Social Studies at the junior high school level, whose goal is to help students comprehend social phenomena and acquire essential life skills for building a better, more adaptive society amid global change. According to *BSNP Regulation No. 033 of 2022*, IPS education aims to cultivate critical thinking, communication, creativity, and collaboration within a civic and environmental framework. These competencies can only be achieved through innovative pedagogies that contextualise knowledge with real-world social and environmental challenges. Integrating Minangkabau indigenous knowledge into social studies learning provides a meaningful and contextual approach to disaster education. Minangkabau culture contains abundant ecological and moral values that support environmental stewardship and resilience. Examples include the architectural design of the Rumah Gadang, which employs flexible wooden pegs to withstand earthquakes; the *rimbo larangan* [sacred forest conservation]practice, which preserves ecological balance; and traditional proverbs such as *alam takambang jadi guru* [‘nature as the ultimate teacher’], which instil respect for natural systems. These values reflect a holistic understanding of the interdependence between humans and the environment, a foundation that aligns perfectly with disaster risk reduction principles. Oral traditions such as Rabab Pasisia, a form of poetic storytelling accompanied by a string instrument, also play a vital role in transmitting disaster-related wisdom. Performances like Rabab Pasisia: Banjir di Solok Selatan convey narratives about natural hazard, urging attentiveness to environmental signs, fostering social cohesion, and reinforcing ecological ethics. Similarly, myths such as the prohibition against building houses on *tanah baranak galodo* [flood-prone land] or mining rivers for gold encode environmental warnings within moral narratives. These cultural forms function not only as entertainment but also as informal educational systems that teach risk perception and community adaptation through collective memory. Integrating these local traditions into social studies learning ensures that disaster education is both culturally relevant and pedagogically effective. Classroom projects may include documenting oral histories, analysing local proverbs for environmental meaning, or simulating community-based disaster preparedness rooted in Minangkabau customs. Such approaches not only strengthen students’ disaster literacy but also preserve cultural heritage, creating a dual benefit of education and cultural sustainability. The model proposed in this study corresponds with international policy frameworks such as the Sendai Framework and UNESCO’s Culture-Based Education Recommendations, both of which emphasise that disaster resilience must be rooted in cultural identity and local participation. Therefore, the integration of indigenous knowledge into education not only meets national curriculum requirements but also contributes to the global agenda for sustainable and inclusive disaster risk reduction. Given these contexts, this research addresses two fundamental questions: (1) What is the potential of Minangkabau indigenous knowledge as a source of values, knowledge, and socio-cultural practices that can be integrated into social studies (IPS) learning to support disaster mitigation efforts in West Sumatra?; (2) How can a pedagogical model be developed to integrate Minangkabau indigenous knowledge into contextual, meaningful social studies instruction aligned with the Merdeka Curriculum, the Sendai Framework, and UNESCO’s recommendations? Through these inquiries, the study seeks to identify specific elements of Minangkabau indigenous knowledge that can enhance the effectiveness of disaster education in junior high schools, particularly within the Grade 7 IPS curriculum. The ultimate goal is to develop a culture-based disaster mitigation model that simultaneously builds students’ resilience, strengthens their cultural identity, and supports the realisation of global disaster risk reduction objectives.

## Research methods and design

The data collection process, aimed at addressing the research objective of identifying Minangkabau indigenous knowledge values related to the disaster sub-theme in Grade 7 social studies, was carried out in six main stages. Firstly, a Focus Group Discussion (FGD) was conducted with social studies teachers across West Sumatra to gather insights on curriculum integration and local contextualisation. Secondly, another FGD was held with Minangkabau cultural and traditional leaders from various regions in West Sumatra to explore indigenous perspectives on disaster-related wisdom. Thirdly, a library research phase was undertaken to review existing literature, previous research findings, and official documents from institutions such as BNPB, UNESCO, United Nations Office for Disaster Risk Reduction (UNDRR), and the Ministry of Education, Culture, Research, and Technology (Kemendikbudristek), as well as local Minangkabau sources relevant to disaster mitigation based on traditional knowledge. Fourthly, field observations were conducted to identify and document manifestations of Minangkabau cultural values related to disaster preparedness in various local communities across West Sumatra. Fifthly, in-depth interviews were conducted purposively with selected key informants, including social studies teachers, traditional leaders, and officials from BNPB and BPBD. These interviews were intended to elicit a deeper understanding of the underlying philosophical and practical dimensions of Minangkabau cultural wisdom in disaster mitigation. Sixthly, documentation was used to collect relevant materials, such as curriculum documents, ministerial regulations, teacher and student textbooks, teaching modules, and visual and archival evidence, including photographs, field notes, interview recordings, and traditional Minangkabau manuscripts or proverbs. These six stages complemented each other, ensuring triangulated data that provided a comprehensive and valid portrayal of the research focus. We employed purposive sampling to select informants who met criteria aligned with our research objectives. These criteria ensured the collection of valid, detailed, and relevant data regarding Minangkabau indigenous knowledge in disaster mitigation for Grade 7 social studies. A total of 18 informants participated in the study and were organised into three categories; each was assigned a specific informant code. The first group consisted of eight social studies teachers from junior high schools (SMP or MTs) in West Sumatra, labelled SST1–SST8. These teachers were selected based on their experience teaching Grade 7 social studies, a minimum of 5 years of teaching experience, prior instruction of the ‘Disasters in Indonesia’ sub-theme, and their familiarity with Minangkabau culture. The second group comprised eight Minangkabau traditional leaders, labelled TL1–TL8, representing different Nagari and diverse customary backgrounds. These leaders hold respected positions within the Minangkabau community, actively preserve local traditions, possess at least 10 years of experience, and demonstrate extensive knowledge of indigenous knowledge and traditional perspectives on disasters. The third category comprised two disaster management officials from BNPB/BPBD, labelled DMO1 and DMO2, who serve at the provincial and district or city levels in West Sumatra. These officials were selected for their roles in disaster management, practical experience in disaster mitigation or community-based risk reduction, and at least 3 years of professional experience. Including these three groups enabled us to compare perspectives across educational, cultural, and institutional domains, thereby enhancing the credibility and depth of the study’s analysis (Sugiyono 2015). Data analysis in this study employed the interactive model developed by Miles, Huberman and Saldana (2014), which consists of three interconnected stages: data reduction, data display, and conclusion drawing with verification. In the data reduction stage, information obtained from FGDs with teachers and traditional leaders, observations, interviews, library research, and documentation were carefully sorted, categorised, and focused on the central theme of the study, namely, the integration of Minangkabau indigenous knowledge into social studies learning as a strategy for disaster mitigation. Irrelevant data were excluded to maintain analytical clarity and direction (Miles et al. 2014). In the subsequent stage, data display, the reduced information was organised into descriptive narratives, tables, matrices, and diagrams to facilitate the identification of emerging patterns, interrelationships, and tendencies, particularly regarding how local cultural values are integrated into the learning process and how they contribute to disaster mitigation efforts. The final stage, conclusion drawing and verification, involved formulating preliminary conclusions based on observed patterns and then re-examining them through data triangulation to ensure the validity and reliability of findings. This verification process was conducted continuously throughout the study to ensure that the conclusions are credible, consistent, and accountable (Bungin 2017; Miles et al. 2014). This analytical approach provided a comprehensive understanding of the values of Minangkabau indigenous knowledge integrated into social studies education, revealing their vital contribution to fostering disaster awareness and preparedness among communities in West Sumatra.

### Ethical considerations

Ethical clearance to conduct this study was obtained from the Universitas PGRI Sumatera Barat Research Ethics Committee (Ref. No. 113/UPGRISBA-PPM/X/2025). The research strictly followed ethical principles consistent with qualitative research standards in the social sciences. Informed consent was prioritised by clearly explaining the study’s objectives, benefits, and procedures to all participants, ensuring voluntary involvement without coercion. Confidentiality was maintained through anonymisation and the use of coded identifiers, safeguarding the privacy and safety of all informants. The principle of non-maleficence was upheld by ensuring that no harm was done to participants or their communities, and that all data collection was conducted respectfully in accordance with Minangkabau cultural norms. The study also emphasised beneficence by contributing academically, pedagogically, and socially to the improvement of indigenous knowledge-based disaster mitigation practices in West Sumatra. Additionally, the principle of justice was implemented by treating all participants equally, regardless of age, gender, or social background. The researcher maintained honesty, transparency, and integrity in reporting, ensuring that the findings accurately reflected field realities without manipulation. Thus, this study not only pursued academic rigour but also embodied moral responsibility toward research participants and the local communities involved (Creswell 2019).

## Results

Indonesia’s geographical position at the convergence of three major tectonic plates makes the country highly vulnerable to earthquakes, tsunamis, volcanic eruptions, and other geological disasters. This vulnerability is also evident in the province of West Sumatra, which faces a high potential for such natural hazards. In this context, the wisdom and cultural values of the Minangkabau people play a crucial role in disaster mitigation efforts. Through values, traditions, and local knowledge passed down across generations, the Minangkabau community has developed adaptive mechanisms that enable it to survive and reduce the risks associated with natural hazards. Interviews reveal that Minangkabau indigenous knowledge actively guides adaptive strategies for effectively managing natural disaster risks in West Sumatra.

### Natural disaster characteristics in West Sumatra

#### Earthquake

**The architecture of Rumah Gadang as representation of Minangkabau knowledge:** The findings of this study reveal that the Minangkabau people possess a sophisticated and deeply rooted system of traditional knowledge in responding to earthquakes, one of which is clearly manifested in the architectural design of the Rumah Gadang. West Sumatra lies in an area of intense seismic activity due to the convergence of the Indo-Australian and Eurasian Plates, which frequently triggers tectonic earthquakes. A significant example is the 7.6-magnitude earthquake that struck on 30 September 2009, causing extensive damage across Padang and surrounding regions (Damsar & Indrayani 2018). In addition to tectonic activity, volcanic earthquakes are common near Mount Marapi and Mount Talang, further highlighting the region’s high exposure to seismic hazards. These recurring events have shaped the collective knowledge and adaptive strategies of the Minangkabau community, influencing both cultural practices and architectural innovations.

Interviews with traditional experts, such as Mr ZK and Mr FD (customary authority from Tanah Datar and Kota Padang), revealed that the Rumah Gadang architecture embodies a sophisticated form of environmental adaptation to an earthquake-prone region. The house’s vertical pillars are not directly embedded into the ground but rest upon broad, flat stones that serve as flexible foundations. This design enables the entire structure to sway naturally in harmony with seismic waves, minimising structural damage during earthquakes. The slightly outward-leaning pillars, symbolically described as resembling a ship sailing on turbulent seas, reflect the Minangkabau philosophy of flexibility, endurance, and balance in facing natural disruptions. Another remarkable technical adaptation involves the use of wooden pegs (pasak kayu) instead of metal nails. This method allows the interlocking wooden beams to shift and absorb seismic vibrations, creating an elastic yet stable structure. The Rumah Gadang also features side projections known as anjung, built without supporting pillars to balance the house’s central frame. The symmetrical tension between both sides reinforces structural integrity, allowing the building to maintain equilibrium during tremors. This architectural system demonstrates a high level of indigenous engineering that aligns with modern seismic design principles. Here is the form of Rumah Gadang.

**FIGURE 1:**
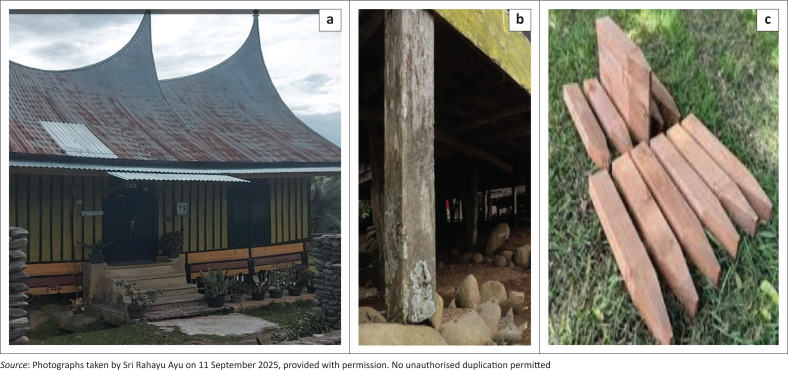
(a) Architectural form of the Rumah Gadang in West Sumatra; (b) Structural pillars of the Rumah Gadang; (c) Wooden pegs (pasak kayu) used in the traditional construction of the Rumah Gadang.

Figure 1 illustrates the functional role of wooden pegs (pasak kayu, cylindrical wooden fasteners used to join building components) during seismic events. As shown in Figure 1, the wooden pegs respond flexibly, moving towards the supporting pillars, allowing the building components to dynamically adjust to ground motion. This synchronised movement creates a uniform structural rhythm that dissipates seismic energy and reduces stress concentration within the building framework. As a result, the Rumah Gadang maintains its structural integrity and demonstrates a high resistance to collapse during earthquakes. In addition, the left and right sides of the house feature projecting structures known as *anjung* [side extensions], which are constructed without supporting pillars. These elements play a crucial role in maintaining structural balance during ground shaking. The symmetrical tensile forces (pulling forces that attempt to elongate a structural member) generated from both sides enhance the strength of the central frame, enabling it to withstand vertical loads more effectively under seismic conditions. The field observations correspond closely with findings from Saptiningtyas, Paturusi and Dwijendra (2023), who confirmed that traditional Rumah Gadang construction effectively absorbs seismic energy and can serve as a valuable reference for modern earthquake-resistant housing design (Saptiningtyas et al. 2023). Beyond its physical architecture, Minangkabau indigenous knowledge also encompasses socio-cultural mechanisms of disaster awareness. The study, ‘*Reading indigenous signs: The wisdom of nagari communities toward natural disasters in Pasaman Barat*’, demonstrates how rural communities interpret environmental signals, such as changes in animal behaviour, wind direction, and atmospheric phenomena, as early warning indicators. This semiotic system reflects deep ecological intelligence and provides evidence that indigenous knowledge operates as a living, adaptive knowledge system. In connection with the present research, ‘*Disaster mitigation based on Minangkabau indigenous knowledge in social studies learning*’, these findings affirm the importance of incorporating traditional ecological knowledge into formal education. Teaching students how Minangkabau communities ‘read’ natural signs fosters disaster literacy rooted in cultural identity. It not only enriches social studies learning but also nurtures ecological empathy, moral reasoning, and critical thinking, key competencies for developing disaster-resilient future generations (Nopriyasman et al. 2024). As Rozi et al. (2021) explains, Minangkabau indigenous knowledge integrates cognitive, social, and moral dimensions that can strengthen community-based disaster response. This provides a strong conceptual foundation for embedding indigenous knowledge into education (Rozi et al. 2021). In this context, the Rumah Gadang serves as a tangible symbol of how indigenous architectural and cultural practices embody disaster mitigation strategies. Integrating these values into Grade 7 social studies learning, particularly within the sub-theme ‘Disasters in Indonesia’, can cultivate students’ appreciation of cultural heritage while instilling awareness of safety, sustainability, and resilience in harmony with nature.

#### Memories of past disasters (oral traditions)

The findings reveal that the Minangkabau people possess a rich oral tradition that effectively transmits disaster-related knowledge across generations. Through storytelling, advice, and lived experience, the community preserves a collective memory of earthquakes, fostering vigilance and preparedness in daily life. These oral traditions serve not merely as historical recollections but as social education that promotes safety and environmental adaptation. For instance, in West Pasaman, Mr OZ, the customary authority from Pasaman) recounted how stories about the 1977 earthquake are still told by parents to their children. The repeated quakes that year encouraged simple adaptive practices, such as securing furniture to walls, an enduring example of community-based mitigation. Similarly, according to Mr RZ customary authority), from Kampung Tinggam, Nagari Kajai, traditional Rumah Gadang and stilt houses arranged along valleys successfully withstood the 2022 earthquake, reinforcing local confidence in ancestral architectural wisdom. These oral narratives demonstrate that Minangkabau indigenous knowledge embodies both symbolic and practical approaches to disaster resilience. They cultivate values of vigilance, cooperation, and reverence for ancestral knowledge as life principles. In Grade 7 social studies, such traditions can serve as contextual learning resources that help students identify disaster signs, appreciate cultural heritage, and foster environmental responsibility, thereby bridging indigenous wisdom and modern disaster education.

#### Traditional proverb

The findings of this study reveal that *petatah-petitih* [traditional proverbs] are a profound embodiment of Minangkabau indigenous knowledge, functioning as tools for moral, social, and ecological education (Fitri et al. 2025). These proverbs convey layered meanings through metaphors and indirect expression, reflecting a communicative culture that values subtlety and reflection. This aligns with the findings of Febraningsih et al. (2022), who emphasise that petatah-petitih transmit moral reasoning, cultural identity, and communal ethics among younger generations. In the context of disaster mitigation, petatah-petitih function as repositories of ecological wisdom, teaching people to live harmoniously with nature (Febraningsih et al. 2022). Proverbs such as ‘*Alam takambang jadi guru*’ [‘Nature unfolds as a teacher’] and ‘*Ingek-ingek nan di ateh, nan di bawah kok maimpok*’ [‘Be cautious of what comes from above and below’] encourage vigilance toward natural hazards. Integrating *petatah-petitih* into social studies learning nurtures environmental awareness and disaster preparedness among students.

#### Tsunami

The study by Harahap, Masselink and Boulton (2025), titled ‘*A Coastal Risk Analysis for the Outermost Small Islands of Indonesia: A Multiple Natural Hazards Approach*’, underscores the importance of understanding multi-hazard risks, coastal and geophysical, in improving the resilience of Indonesia’s small island communities. Their research shows that vulnerability and exposure levels differ across regions, influenced by environmental and socio-economic factors, and thus require localised adaptation and disaster education. This aligns with the present study, ‘*Disaster mitigation based on Minangkabau indigenous knowledge in social studies learning*’, which integrates cultural and ecological knowledge into education to enhance preparedness. While Harahap et al. emphasise quantitative hazard mapping, this study focuses on socio-cultural dimensions, revealing how indigenous systems such as *petatah-petitih*, tambo, and settlement patterns serve as qualitative resilience mechanisms (Harahap et al. 2025). Together, both perspectives promote sustainable disaster mitigation through scientific analysis and cultural empowerment rooted in indigenous knowledge.

#### Interpreting natural signs

The indigenous knowledge of the Minangkabau people in responding to tsunami threats is reflected in their traditional ecological knowledge and spatial awareness, which function as a natural early warning system. Guided by the philosophy ‘*Alam takambang jadi guru*’ [‘Nature unfolds as a teacher’], Minangkabau communities have long learned from environmental cues such as unusual bird movements, sudden sea-level changes, or the behaviour of marine animals to anticipate natural hazards. Traditionally, settlements were established in highland areas, far from the coastline, reflecting a deep ecological understanding aligned with modern disaster risk reduction principles. This wisdom parallels the findings of Esteban et al. (2013), who emphasised that knowledge, attitudes, and practices strongly influence community preparedness in tsunami-prone regions. Field interviews further revealed that after the 2009 earthquake, villagers instinctively observed sea and animal behaviour to assess tsunami risk. Such practices demonstrate how local knowledge fosters vigilance, environmental harmony, and resilience, illustrating the enduring relevance of indigenous ecological literacy within sustainable disaster mitigation frameworks in Indonesia (Esteban et al. 2013).

#### Traditional proverb

Petatah-petitih represent verbal indigenous knowledge in Minangkabau culture, rich in meaning and expressed through metaphorical and indirect language. In tsunami mitigation, the proverb ‘*Jikok takuik dilamun ombak, jan barumah di tapi pantai*’ [‘If you fear being swept by the waves, do not build a house by the shore’] functions as both an ecological and social warning, advising people to avoid settling in disaster-prone coastal areas. It underscores the importance of spatial awareness and environmental prudence. Pedagogically, this proverb offers a valuable learning tool that cultivates critical thinking, ethical reflection, and environmental responsibility among students. Hasanadi’s (2018) study on indigenous knowledge in Pasaman Barat emphasises that petatah-petitih serve as cultural mechanisms embedding moral, ecological, and ethical values, guiding social behaviour and fostering collective environmental awareness within the Minangkabau community (Hasanadi 2018).

### Practising tradition [*Hoyak Tabuik*]

The *Hoyak Tabuik* tradition in Pariaman embodies indigenous knowledge that functions as a spiritual and social mechanism for mitigating maritime disasters. Celebrated annually in Muharram, it involves a community-wide procession that culminates in the casting of the tabuik, a symbolic effigy, into the sea to release misfortune [*bala*] and safeguard the community. According to interviews, this tradition has been preserved across generations, attracting visitors from various regions while maintaining its sacred meaning. Beyond its religious aspect, Hoyak Tabuik strengthens social cohesion, cultural identity, and contributes to local tourism and economic welfare. Akmal and Lubis (2022) emphasise that the ritual, rooted in Minangkabau oral and religious heritage, symbolises moral, spiritual, and ecological harmony. Through the act of casting the tabuik into the sea, the community collectively enacts purification and balance between humans and nature, reflecting integrated resilience and faith-based disaster awareness (Akmal & Lubis 2022).

### Mangrove and casuarina tree planting in coastal areas

The coastal communities of Pariaman cultivate mangroves and casuarina trees along the shoreline to protect against tsunamis and coastal erosion. Rooted in local beliefs, this practice is viewed as an environmental virtue that wards off disasters while sustaining ecological balance. These trees form a natural green belt, absorbing wave energy, reducing tsunami currents, and preventing shoreline abrasion. Examples of mangrove vegetation along the Pariaman coastline, illustrating coastal protection and ecosystem-based disaster mitigation.

**FIGURE 2:**
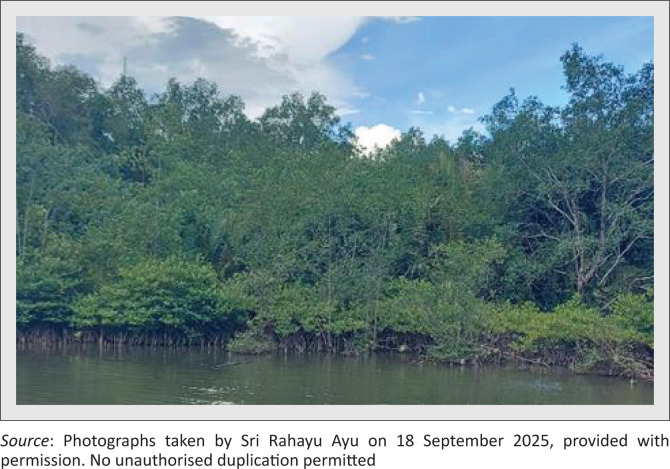
Mangrove Park in Apar, Pariaman.

As shown in Figure 2, these plant species serve as a natural green belt that attenuates wave energy, reduces tsunami current velocities, and prevents coastal erosion. This practice reflects strong ecological awareness and the collective responsibility of local communities toward environmental conservation. Beyond their ecological role, mangrove planting activities embody communal cooperation (gotong royong) and shared environmental stewardship. In recent years, these initiatives have been systematically integrated into local government programmes and ecology-based tourism development, such as the Apar Pariaman Mangrove Park, thereby enhancing not only environmental protection but also socio-economic resilience. As noted by Harto et al. (2021), such forms of indigenous knowledge contribute significantly to disaster education through the integration of cultural values, pedagogical practices, and community participation, key dimensions for fostering environmental awareness and sustainability in social studies learning (Harto et al. 2021).

### Volcanic eruption

A volcanic eruption occurs when high-pressure gases within the Earth’s crust force magma to the surface, releasing molten rock, ash, and hot gases exceeding 1000°C. Mount Merapi in West Sumatra, one of Indonesia’s active volcanoes, functions not only as a site of geological activity but also as a living laboratory for cultivating indigenous knowledge in disaster mitigation. Communities surrounding Merapi have long relied on traditional knowledge to detect early warning signs, such as changes in sulphur odour, underground rumblings, and the descent of animals from the slopes. Through gotong royong, they built Rumah Gadang stilt houses resilient to earthquakes and safe from cold lava flows. The Minangkabau philosophy *alam takambang jadi guru* [‘nature unfolds as a teacher’] reflects how people learn from nature’s patterns. As highlighted by Anwar (2021), this indigenous knowledge embodies environmental ethics, cooperation, and adaptive behaviour, forming a cultural foundation for disaster mitigation learning in social studies education (Anwar 2021).

### Reading natural signs

The ability to interpret natural signs has been passed down through generations as a vital form of indigenous knowledge. Communities observe various environmental indicators, such as a sudden rise in temperature, changes in the colour of the sky, and changes in animal behaviour, including roosters ceasing to crow and dogs barking continuously. In certain regions, the appearance of *inyiak* [Sumatran tigers] or the descent of *uyia-uyia* [forest beetles] from mountain slopes is believed to signal extraordinary natural events. In West Pasaman, people also observe cloud formations around Mount Talamau; when the clouds form a ring and gradually descend, it is interpreted as a sign of impending disaster. This traditional ecological knowledge reflects a deep spiritual and ecological relationship between humans and their environment, reinforcing community preparedness and resilience in the face of natural hazards.

### Selection of safe settlement locations

The Minangkabau community demonstrates indigenous knowledge in selecting settlement sites. Traditional houses and ancient *nagari* [villages] around Mount Marapi, such as those in Batu Palano and Koto Baru, are strategically built on moderate to high ground, which is relatively safe from lava flows and landslides. This practice reflects an ecological awareness that balances safety considerations with sustainable living. In addition to avoiding high-risk zones, these locations maintain access to fertile agricultural land and clean water sources. Therefore, this spatial wisdom functions not only as a disaster mitigation strategy but also as a means of preserving social and economic sustainability within the community.

### Customary prohibitions and traditional regulations

Indigenous knowledge is also embodied in customary rules that prohibit land clearing near volcanic areas. These zones are known as *rimbo larangan* [sacred or forbidden forests], which are ancestrally protected customary forests. The rimbo larangan functions as an ecological buffer, preventing erosion and lahar flooding, while also serving as a safety zone from potential volcanic hazards. Beyond their ecological role, these sacred forests hold social and spiritual significance. Violating the taboo of opening or exploiting these areas is believed to invite misfortune or natural disaster. The findings of Imamulhadi et al. (2025) on customary environmental law and its transformation models in Indonesia emphasise that customary environmental law in Indonesia functions as a living legal system that regulates the relationship between humans and nature through moral, social, and ecological values. This aligns closely with the concept of disaster mitigation based on Minangkabau indigenous knowledge, which also relies on customary norms [*adat basandi syarak, syarak basandi Kitabullah*] as guiding principles in maintaining environmental balance. Both frameworks highlight the continuity of indigenous law as a dynamic and adaptive system that contributes to environmental protection and risk reduction (Imamulhadi et al. 2025).

### Myth

On the western slopes of Mount Merapi lies Batu Anguih, a sacred site believed to hold profound spiritual significance. According to local oral tradition, the stone marks the place where a revered ancestor, Inyiak, prayed for divine protection during a great eruption. It is said that his prayers caused the lava to stop at the stone, sparing the surrounding village from destruction. This myth reflects how the Minangkabau people interpret natural phenomena through symbolic and spiritual narratives, blending faith and environmental awareness. Though mystical in nature, such stories serve important educational and moral functions by promoting vigilance, gratitude, and harmony with the environment. In line with this, Mangunjaya and McKay (2012) emphasise that Islamic environmental ethics, grounded in the principles of *khalifah* [stewardship] and *amanah* [moral responsibility], can strengthen ecological awareness. Environmental preservation, therefore, is not only a scientific effort but also a sacred moral obligation within cultural and religious contexts (Mangunjaya & McKay 2012).

### Flash floods (Galodo)

Flash floods, or galodo, are among the natural hazards that frequently occur in the Minangkabau region, particularly in areas with steep slopes and river valleys. The term galodo refers to a large-scale flood that carries mud, rocks, and logs and occurs suddenly due to extreme rainfall or upstream landslides. This phenomenon is also known as a debris flow or rain-induced lahar, characterised by its high destructive power and often resulting in loss of life and severe environmental damage. According to Madinier (2015), flash floods have a recurrence period of several decades in a given area. They are typically accompanied by fine sediments and large boulders carried by the current. The Minangkabau people have long developed various forms of indigenous knowledge to identify, anticipate, and minimise the impacts of such disasters. The following are the manifestations of Minangkabau indigenous knowledge in responding to galodo (Madinier 2015).

### Reading natural signs

Before the emergence of modern early warning systems, the Minangkabau people possessed traditional ecological knowledge that enabled them to interpret natural signs as indicators of impending *galodo* [flash floods]. This indigenous wisdom, preserved through oral traditions and continuous environmental observation, reflects a deep understanding of nature’s signals. Common indicators include changes in river water colour when it turns dark brown and carries twigs or leaves, suggesting landslides or heavy rain upstream. Rumbling sounds from the upper river, especially at night after heavy rain, indicate that large objects, such as rocks or logs, are being swept downstream. Unusual animal behaviour, such as birds, monkeys, or snakes descending from forests, signals ecological disturbance. Additionally, dark clouds over the highlands and humid, cold air often foretell extreme rainfall. This is captured in the proverb ‘*Awan hitam di hulu, batang barubah di hilia*’ [‘When dark clouds gather in the highlands, the river downstream will change’], symbolising ancestral risk communication that remains vital for disaster education today.

### Selecting safe settlement locations

The indigenous knowledge of the Minangkabau people is evident in their traditional methods of selecting settlement sites that minimise the risk of flash floods. Site selection was guided by careful observation of land position, surface contours, and water flow direction. According to field interviews, Mr S (customary authority explained that the Minangkabau ancestors were highly cautious in choosing residential areas, avoiding valleys or riversides in line with the principle ‘*jangan lawan alur aia*’ [‘never go against the direction of water flow’]. Mr L (customary authority noted that areas known as *tanah larangan* or *tapian aia gadang* were recognised as dangerous zones where strong currents once passed, marked by the presence of large rocks or logs. Mrs. S (a social studies teacher) added that elders often advised, ‘*carilah tanah nan manjadi tempat aia manyiarik*’ [‘choose land where water flows gently’], as violating natural laws could bring disaster. This wisdom illustrates an adaptive, eco-conscious tradition that effectively mitigates disaster risks.

### Traditional proverb

Minangkabau indigenous knowledge is also embedded in petatah-petitih traditional sayings, which are rich in ecological and mitigative meanings. One of the most notable is the philosophy ‘*Aua jo tabiang sanda basanda*’. This expression symbolises the reciprocal relationship between bamboo and riverbanks, each supporting the other in resisting erosion. The bamboo strengthens the riverbank, while the riverbank provides a firm base for the bamboo to grow. This philosophy reflects the Minangkabau people’s ecological awareness of the importance of preserving vegetation along river basins (Daerah Aliran Sungai or DAS) to reduce the risk of *galodo* [flash floods]. Such values serve as a foundation for environmental education among younger generations, emphasising the importance of maintaining natural balance.

### Customary prohibitions and traditional regulations

Customary prohibitions [*larangan adat*] are a vital component of the Minangkabau social system, guiding community behaviour to remain in harmony with nature. Though unwritten, these norms possess strong moral authority. One important rule forbids building houses on tanah larangan, former galodo paths, or flood-prone areas. Violations are traditionally addressed through *musyawarah adat* [customary deliberation]. Another prohibition warns against cutting trees on hill slopes, as expressed in the maxim: ‘*Jangan manyabik bukik, jangan manaruko rimbo di tampak aia turun*’, meaning ‘Do not cut the hill or clear the forest where water flows’. According to Mr J (a social studies teacher), breaking this rule was believed to anger ‘the water’, a notion now validated by science, as deforestation increases the risk of flash floods. Such prohibitions embody ecological wisdom embedded in culture. As noted by Hutagalung and Indrajat (2020), Indonesia’s local knowledge systems through taboos, rituals, and community education effectively strengthen disaster resilience and sustainable environmental management (Hutagalung & Indrajat 2020).

### Myths

Myths in Minangkabau culture serve not only as folklore but also as educational tools that transmit moral values and environmental awareness. Though lacking scientific proof, they are accepted as truth and function as guides for harmonious living with nature. In Nagari Sitalang, Agam Regency, for instance, a myth warns against building houses on *tanah baranak galodo* land that has been struck by flash floods. Based on interviews with a traditional leader [*datuak*] who plays an important role in preserving customary traditions and transmitting local knowledge within the community, Dt. Rajo Malano reports that violating this taboo is believed to anger the ‘spirit of nature’, triggering another disaster. Consequently, the community continues to avoid settling in these high-risk areas. A similar belief exists in Nagari Tanjung Gadang, Sijunjung Regency, where residents refrain from panning for gold in sacred rivers, fearing misfortune. This practice helps preserve river ecosystems and prevent environmental degradation. These myths demonstrate that indigenous knowledge in Minangkabau society serves as an effective, culturally rooted approach to disaster mitigation, fostering respect for nature and sustainable living.

### Rabab

Rabab Pasisia is a traditional Minangkabau performing art originating from the coastal areas of South Pesisir, West Sumatra. It combines music and storytelling through poetic singing accompanied by the rabab, a traditional string instrument. The performer narrates tales of community life, legends, and moral lessons, transforming artistic expression into a form of cultural communication. Beyond entertainment, Rabab Pasisia carries messages of environmental awareness and disaster preparedness. A notable example, ‘*Rabab Pasisia: Banjir di Solok Selatan*’ by Prin Jambak and Gadih, recounts a devastating flood from heavy rainfall to river overflow – emphasising attentiveness to natural signs and the importance of environmental conservation. Through this narrative, Rabab serves as an educational medium that fosters vigilance, ecological awareness, and collective responsibility. Khusairi and Nurhayati (2025) highlight that traditional arts, such as the Kaba Festival in West Sumatra, act as channels of moral instruction and ecological reflection. Through poetic storytelling and musical expression, they preserve collective memory while reinforcing community resilience against natural and social challenges. The researchers note that the communicative power of oral traditions lies in their ability to evoke emotional and intellectual engagement, making them effective tools for cultural education (Khusairi & Nurhayati 2025). Aligned with this perspective, Rabab Pasisia demonstrates how indigenous knowledge can be integrated into Social Studies learning under the theme of ‘Disaster Mitigation Based on Minangkabau Indigenous Knowledge’. By incorporating these art forms into education, students can develop empathy, environmental literacy, and disaster awareness, linking cultural heritage with sustainable, character-based learning.

### Integration of indigenous knowledge in disaster mitigation education

In the context of disaster vulnerability, the study demonstrates that Minangkabau indigenous knowledge is not adequately integrated into disaster education. Evidence from classroom observations, key informant interviews, and instructional document analysis collectively indicates that local knowledge remains marginal within social studies instruction. Data collection involved classroom observations of Grade 7 social studies classes, in-depth interviews with informants, and analysis of curriculum materials. Observation lessons on natural hazards in junior high schools in West Sumatra revealed limited integration of Minangkabau indigenous knowledge. Most teachers employed a descriptive, textbook-centred approach that emphasised disaster types, causes, and impacts. Local knowledge was introduced informally as supplementary examples rather than systematically embedded in lessons, underscoring a gap in the curriculum’s incorporation of indigenous knowledge. A Grade 7 teacher (YS) reported that while textbook materials address disaster types and impacts, examples from Minangkabau culture are seldom included and are typically communicated verbally. Another teacher (SB) noted that incorporating local folklore could enhance student engagement, although such content is not included in official modules. Interviews with Minangkabau customary leaders indicate that disaster mitigation values are deeply embedded within local traditions. One informant (FD) stated, ‘Elders warned that areas flooded in the past should not be resettled, because nature remembers’ (customary leader, datuak). This local knowledge functions as an early warning system grounded in historical experience. Interviews with Regional Disaster Management Agency (BPBD) officials suggest that modern mitigation principles are consistent with community practices. A BPBD official stated that identifying hazard zones, restricting riverbank use, and preserving river ecosystems are essential mitigation strategies, as reflected in Minangkabau customary regulations. This demonstrates compatibility between traditional and contemporary disaster mitigation approaches. Document analysis of the Grade 7 social studies curriculum and textbooks reveals two primary findings: disaster-related content is formally included in the national curriculum, but references to indigenous knowledge are general and lack specific contextualisation within Minangkabau culture. Teaching modules and textbooks do not explicitly incorporate local myths, oral traditions, or traditional arts such as Rabab Pasisia as educational resources. This insufficient contextualisation represents a significant missed opportunity to position indigenous knowledge as a central component of disaster mitigation education.

## Discussion

The indigenous knowledge of the Minangkabau people illustrates how cultural values and traditions can serve as an integral part of a disaster mitigation system passed down through generations. One manifestation of this wisdom is found in myths and folk tales believed to have truly occurred that convey moral lessons and life guidance for the community. In the context of disaster mitigation, myths function as a form of cultural communication that instils collective awareness of potential hazards in a given area. As recounted by Dt. Rajo Malano (customary authority ‘datuak’) from Nagari Sitalang, Agam Regency, there is a myth prohibiting people from building houses on land known as tanah baranak galodo. The community believes that massive floodwaters once traversed this area, and any violation would provoke the ‘spirit of nature’ to unleash another flash flood. This belief is not merely superstition; it reflects ecological knowledge encoded in symbolic forms that the community readily accepts and remembers. Such myths serve as a local early- warning system, encouraging residents to recognise hazard-prone areas through culturally resonant narratives. These findings are consistent with the studies by Adger et al. (2013) and Gaillard and Mercer (2013), which demonstrate that traditional ecological knowledge often embodies empirical information concerning disaster-prone zones, albeit articulated through symbolic language and culturally embedded belief systems. In this sense, indigenous knowledge can serve as an informal early warning system for disaster mitigation. A similar myth exists in Nagari Tanjung Gadang, Sijunjung Regency, where locals are forbidden from panning for gold in certain rivers, as it is believed to bring death. This prohibition carries an ecological message, discouraging the community from disrupting the natural balance. By refraining from exploiting the river, residents indirectly preserve water quality and reduce flood risk caused by ecological damage. These examples demonstrate that myths are not merely irrational old tales; they function as instruments of ecological education and culturally grounded disaster mitigation.

His evidence indicates a close interrelationship between Minangkabau traditional ecological knowledge and modern disaster mitigation science. Local knowledge is not merely symbolic; it aligns conceptually with contemporary disaster risk reduction principles. For example, the prohibition against establishing settlements in *tanah baranak galodo* [landslide-prone areas] corresponds with hazard mapping and risk-based spatial planning in modern disaster science. These approaches both emphasise avoiding dangerous areas, drawing on historical experience and systematic risk assessment (Wisner & Adams 2002).

Similarly, disasters are interpreted as consequences of imbalanced human–nature relations, echoing the social–ecological approach and ecosystem-based disaster risk reduction (Eco-DRR). Eco-DRR conceptualises environmental degradation as a driver of increased disaster risk. This convergence reinforces the view that traditional ecological knowledge is empirically grounded and accumulated through intergenerational experience. Such knowledge can be meaningfully integrated with modern mitigation science (Hiwasaki et al. 2014; Mercer et al. 2010). The link between local knowledge and disaster science provides a firm foundation for developing culturally grounded social studies learning models. This procedure helps students see disaster mitigation as both an abstract scientific concept and a socio-cultural practice based on local community experience. In addition to myths, another form of indigenous knowledge that plays a significant role in disaster education in Minangkabau is Rabab Pasisia, a traditional performing art that combines the Rabab, a bowed string instrument, with narratives or poetic verses that convey folk tales, legends, and accounts of social life. Rabab Pasisia developed in the South Coast region of West Sumatra and is known for its rhetorical and emotional power in delivering moral messages. Through its songs and stories, the Rabab functions as an educational medium and a vehicle for transmitting social values. A notable example relevant to disaster mitigation is ‘*Rabab Pasisia: Flood in Solok Selatan*’, performed by Prin Jambak and Gadih, accessible via YouTube. In this performance, the Rabab narrates how floods struck Solok Selatan, how the community responded, and conveys a moral lesson urging people to remain vigilant and maintain ecological balance. Performances like this not only serve as popular entertainment but also as a form of social awareness, fostering empathy and collective responsibility toward the environment. Through linguistic and musical symbols, Rabab instils the understanding that disasters are not merely acts of fate but often result from human negligence in preserving nature. The rabab tradition teaches the importance of harmony between humans and the environment, in accordance with the Minangkabau philosophy of ‘*adat basandi syarak, syarak basandi Kitabullah*’, customs grounded in Islamic law, which underpins community life, as articulated by one datuak, a customary leader from the southern coastal region. The rabab narrative is not merely a form of entertainment but also serves as a medium of warning. He emphasised that environmental degradation inevitably leads to disaster, a message consistently conveyed through the dendang (traditional chants). Thus, Rabab serves as an effective medium for cultural communication, conveying disaster mitigation values both emotionally and aesthetically.

Both forms of indigenous knowledge, myths and Rabab, illustrate how the Minangkabau people internalise disaster-related values within their cultural system. In the context of Grade 7 social studies learning, with the sub-theme of disasters in Indonesia, these examples can serve as concrete illustrations of the enduring educational relevance of traditional knowledge. Integrate indigenous knowledge into Grade 7 social studies through contextual teaching and project-based learning. These methods match the Kurikulum Merdeka’s focus on contextual learning, character development, and socio-cultural resources. Social studies teachers note the lack of systematic integration of indigenous knowledge, revealing a gap between policy and practice. This shows the need for practical, culturally relevant learning models that teachers can use.

Rahman et al. (2020) confirm that integrating indigenous knowledge into disaster education is constrained by limited guidelines and resources. Respondents’ views underscore the challenge of applying locally based disaster education. Teachers can present local myths as case studies and link them to disaster-prone areas and modern mitigation strategies. Students can complete projects like creating disaster risk maps using indigenous knowledge or writing about messages in Rabab Pasisia. Teachers can use the Tanah Baranak galodo myth to teach students the importance of recognising flood-prone areas and of maintaining the environment to prevent disasters. Meanwhile, a Rabab Pasisia performance can be employed as an interactive learning medium that demonstrates the interconnectedness of art, culture, and the environment. This approach allows students to understand disasters not only from a scientific perspective but also from cultural and moral viewpoints. Indigenous knowledge-based learning such as this helps students develop ecological empathy, instil respect for tradition, and foster disaster-resilient behaviour from an early age. Furthermore, applying indigenous knowledge values, such as myths and Rabab, can reinforce government efforts to build risk-aware communities in accordance with the principles of community-based disaster risk reduction. Integrating culture into disaster education also strengthens national identity and positions local culture as a source of inspiration for environmental and social resilience.

Thus, myths and Rabab function not only as cultural heritage but also as contextualised learning tools that enhance the nation’s character and preparedness to face future disaster challenges. Table 1 shows a more straightforward overview of indigenous knowledge in responding to natural hazards in West Sumatra.

**TABLE 1:** Natural disaster potentials in West Sumatra and Minangkabau culture-based mitigation strategies.

No.	Natural disaster potential in West Sumatra	Forms of Minangkabau cultural indigenous knowledge
1.	Earthquake	*Rumah Gadang* architectural design Disaster memory preserved through oral traditions Traditional maxims [*petatah-petitih*]
2.	Tsunami	Reading natural signs Traditional maxims [*petatah-petitih*] Performing traditional rituals Planting mangrove and pine trees along coastal areas
3.	Volcanic eruption	Reading natural signs Selecting safe settlement locations Customary prohibitions and traditional regulations Myths
4.	Flash flood [*galodo*]	Reading natural signs Selecting safe settlement locations Petatah petitih Customary prohibitions and traditional regulations Myths *Rabab* [traditional musical storytelling]

From Table 1, it can be seen that the Minangkabau community possesses various forms of indigenous knowledge that serve as strategies for natural disaster mitigation. Each type of disaster, such as earthquakes, tsunamis, volcanic eruptions, and flash floods [*galodo*], is addressed through distinct yet interrelated local values, traditions, and indigenous knowledge systems. Based on the research findings, Minangkabau indigenous knowledge shows substantial potential for integration into Grade 7 social studies instruction. This is especially true within the sub-theme Disasters in Indonesia. Integration can be achieved through a culture-based learning approach. These approaches position local knowledge as a contextual resource for learning. As a result, students can understand disasters not just as natural phenomena but also as integral to their communities’ social and cultural experiences. One instructional strategy that can be applied is contextual teaching and learning. For example, teachers can use the Tanah Baranak galodo myth as a case study. This myth helps introduce concepts related to flood-prone areas and risk-based spatial planning. During group discussions, students connect the myth’s messages to scientific explanations, focusing on river flow, sedimentation, and vulnerability to flash floods. In this way, the strategy links traditional knowledge with scientific understanding. In addition, traditional arts like Rabab Pasisia can serve as interactive learning media. For example, teachers can present excerpts of rabab performances that narrate disaster events, and then ask students to identify disaster-related messages in the narratives. Building on this, the activity can become a project-based learning task; teachers may assign students to produce narratives, posters, or short videos on culturally grounded disaster mitigation. This approach enhances cognitive understanding while also fostering ecological empathy and social awareness. The integration of indigenous knowledge can be strengthened through collaboration with local communities. For example, teachers may invite customary leaders to participate as resource persons in classroom learning. This practice aligns with participatory learning principles and also reinforces the *Profil Pelajar Pancasila* [Pancasila Student Profile], especially in the areas of global diversity and mutual cooperation. As a result, indigenous knowledge not only supplements the curriculum but also becomes a foundation for cultivating more inclusive, relevant, and impactful social studies instruction.

## Conclusion

This study affirms that Minangkabau indigenous knowledge is more than a symbolic cultural expression. It is traditional ecological knowledge with tangible disaster mitigation functions. Myths and the tradition of Rabab Pasisia specifically transmit this ecological knowledge. They collectively and sustainably instil risk awareness and ethical principles that govern human–nature relationships. The findings also show a substantive relationship between traditional knowledge and modern disaster mitigation principles. This is evident in the identification of disaster-prone zones, the promotion of ecosystem-based environmental management, and the strengthening of community preparedness. Thus, Minangkabau indigenous knowledge is an alternative knowledge system that complements scientific approaches, not just an irrational practice detached from disaster science. In education, integrating myths and Rabab Pasisia into Grade 7 social studies has significant implications. It helps strengthen contextual and meaningful disaster education rooted in students’ socio-cultural realities. This method enhances students’ understanding of disasters and fosters ecological empathy and social responsibility from an early age. Neglecting local knowledge in education risks undermining effective disaster education. Its systematic incorporation, however, can enhance community resilience and cultivate national character in disaster-prone regions.
